# Disease of the Rectum, Their Diagnosis and Treatment

**Published:** 1873-04

**Authors:** 


*Fistula, Hæmorrhoids, Painful Ulcer, Stricture, Prolapsus, and other Diseases of the Rectum, their Diagnosis and Treatment.* By William Allingham, F. R. C. S., etc. Second Edition. Revised and Enlarged. Philadelphia: Lindsay & Blackiston, 1873. Buffalo: T. Butler & Son.

Mr. Allingham's work has been but a short time before the profession, yet it has come to be looked upon as one of the best works extant npon diseases of the rectum. In the short time which has elapsed since the appearance of the first edition the author has added further to his large stock of experience in the treatment of this class of diseases. His observation of some of the more rare affections of the rectum has been more extended, and his rules for diagnosis and treatment have, therefore, in some instances, been slightly modified.

The book as now presented to the profession will continue to merit in a larger degree than ever the confidence of the profession, and justly deserves a place among the best works published upon diseases of the rectum.

